# Microvascular Guidance: A Challenge to Support the Development of Vascularised Tissue Engineering Construct

**DOI:** 10.1100/2012/201352

**Published:** 2012-04-24

**Authors:** Irza Sukmana

**Affiliations:** ^1^Medical Implant Technology-MediTeg Research Group, Department of Biomechanics and Biomedical Materials, Universiti Teknologi Malaysia, P23 UTM Skudai, Johore, 81310 Johor Bahru, Malaysia; ^2^Department of Mechanical Engineering, University of Lampung, Gedung H lantai 2, Jl. Prof. Soemantri Brojonegoro No. 1, Bandar Lampung 35143, Indonesia

## Abstract

The guidance of endothelial cell organization into a capillary network has been a long-standing challenge in tissue engineering. Some research efforts have been made to develop methods to promote capillary networks inside engineered tissue constructs. Capillary and vascular networks that would mimic blood microvessel function can be used to subsequently facilitate oxygen and nutrient transfer as well as waste removal. Vascularization of engineering tissue construct is one of the most favorable strategies to overpass nutrient and oxygen supply limitation, which is often the major hurdle in developing thick and complex tissue and artificial organ. This paper addresses recent advances and future challenges in developing three-dimensional culture systems to promote tissue construct vascularization allowing mimicking blood microvessel development and function encountered *in vivo*. Bioreactors systems that have been used to create fully vascularized functional tissue constructs will also be outlined.

## 1. Introduction

Tissue engineering is an emerging field. In 1987, the National Science Foundation defined tissue engineering as “an interdisciplinary field that applies the principles of engineering and the life sciences towards the development of biological substitutes that restore, maintain or improve tissue function” [1]. Tissue engineering involve the repair and restoration of various tissue and organ functions, while limiting host rejection and side effects for the patient by delivering cells and/or biomolecules through the use of 3D scaffolds [2].

Although there have been some successes to replace and restore some tissues using tissue engineering approaches, these have been mostly limited to thin or avascular tissues, such as cartilage, skin, or bladder [2, 3]. However, for thick and vascular tissues and for most organs, the lack of a sufficient supply of nutrients and oxygen to growing tissues, as well as the waste removal, represent two important factors that limit the successful development and implantation of engineered tissue constructs [4]. These issues of poor mass transport and mass transfer have often led to the failure of the culture process or even to that of implants.

One possible strategy for creating thick engineered tissue substitutes *in vitro* is to use a bioactive scaffold that allows the guidance of endothelial cells promoting microvessel development in a directional fashion in order to vascularize the tissue construct [5]. It is quite well accepted among the scientific community that prevascularization of tissue construct appears to be one of the most favorable and efficient approaches to address the problem of tissue survival due to a lack of oxygen and nutrient supply [6]. The concept of prevascularization mainly involves the incorporation of endothelial cells into a bioactive scaffold to form a capillary network inside the structure prior to its implantation [7]. This could accelerate the formation of functional microvessels within the core of an implant.

The idea of using prevascularized engineered tissue substitutes was first suggested by Mikos et al. (1993) when comparing the performance of prevascularized tissues to nonvascularized ones. Later, Sakakibara et al. (2002) concluded that prevascularization enhanced the benefits of cardiomyocyte transplantation [6, 8]. More recently, Levenberg et al. (2005) demonstrated that prevascularization improved the performance of skeletal muscle tissue constructs when implanted in mice [9]. Furthermore, studies aiming to induce and control vascularization may also advance general knowledge on the development of therapeutics targeting angiogenesis. Numerous pathological conditions are associated with insufficient oxygen and blood supply [2, 10]. Also, uncontrolled angiogenesis is associated with many diseases, including rheumatoid arthritis, macular degeneration, and tumour growth [11].

Advances in tissue engineering have brought significant knowledge on the mechanisms and parameters related to vascularization and angiogenesis development and blood microvessel network formation [11, 12]. This paper will report status of the current research and development related to cell culture systems designed to promote angiogenesis and vascularization. It is necessary to improve our understanding of the mechanisms behind angiogenesis and to apply that knowledge to guide microvessel growth in order to support the development of artificial organs. Other aspects including the challenges in angiogenesis guidance, assessment of angiogenesis and lumen formation, and the use of bioreactor system to culture vascularized tissue constructs will also be outlined.

## 2. Cell Types and Markers

Cells can be isolated from a patient or donor and seeded into a scaffold to allow cell proliferation and support prevascularization process [6, 13]. Subsequently, these constructs can be implanted in the same patient. After implantation, hopefully endothelial cells would develop connections with the existing blood microvessels of the surrounding tissue, forming a microvascular network and allowing adequate tissue perfusion [13].

When tissues become thicker, cells located more than a few hundred microns (about 200 to 300 *μ*m) from capillaries suffer from a lack of oxygen and nutrients, resulting into necrotic conditions [14]. Currently, the development of thick and complex tissues and organs such as the heart, muscles, kidneys, liver, and lung relies, in part, on the knowledge and ability to stimulate microvascular network formation within tissue constructs. Therefore, knowing how to carry out cell seeding within a scaffold and to perform successful *in vitro* cultures to prevascularize tissue constructs prior to their implantation is highly important. As such, researchers rely on the increasing knowledge related to neovascularization (i.e., vasculogenesis and angiogenesis) process to stimulate vascular network formation within three-dimensional tissue constructs [12, 14].

Blood vessels are composed of several cell types and the main ones are smooth muscle cells (SMCs), pericytes, fibroblasts, and endothelial cells. Smooth muscle cells are found in blood vessels, such as in middle layer (i.e., tunica media) of large and small blood vessels, in lymphatic vessels, uterus, and in the gastrointestinal and respiratory systems. Behind the basic function of vascular SMC in blood vessels, that is, to maintain vessels integrity and support the endothelium, they are highly specialized cells for the regulation of blood vessel diameter, vessel contraction, blood pressure, and flow distribution [15]. Furthermore, SMCs synthesize the connective tissue matrix of the vessel wall, which is composed of elastin, collagen, and proteoglycans. There are some markers to identify smooth muscle cells, including smooth muscle *α*-actin (SM*α*A), smooth muscle myosin heavy chain (SM-MHC) [15], SM22*α*, and calponin [16]. To date, SM*α*A is the most commonly used marker of SMC, which represents up to 70% of the actin population in vascular SMC.

Pericytes are perivascular-specific cells that are associated with capillaries and blood microvessel development. Pericytes have the capacity to differentiate into other cell types, including SMC, fibroblasts, and osteoblasts [17]. During blood microvessel development, the coverage of capillary by pericytes is important for the maturation, remodeling, and maintenance of the vascular system via the secretion of growth factors and/or modulation of the ECM. The important role of pericytes in capillary development *in vitro* has been studied by some researchers [17, 18]. For example, endothelial cells cocultured with pericytes, separated by a cellulose membrane, resulted in inhibition of capillary growth, while when pericytes were in contact or close to endothelial cells, endothelial cell growth and capillary development were observed [19].

Fibroblasts are a cell type mostly found in connective tissues, including blood vessels, cartilage, soft tissues (e.g., dermis), and bone. A connective tissue can be defined as a tissue that wraps, connects, nourishes, and supports all other tissues and organs [20]. Fibroblasts originate from mesenchymal cells—mesenchymal cells are progenitor cells capable to form connective tissues and the lymphatic system [21]. During normal development and wound-healing process of connective tissues, fibroblasts produce fibres and secrete factors to maintain the ECM and provide structural support for the tissues. To date, fibroblasts from human skin or skin fibroblasts have been cocultured with endothelial cells in many angiogenesis studies [18, 19, 22, 23]. Also, 3T3 cells that were originally obtained from Swiss mouse embryo tissues have become a standard fibroblast cell line [18]. In an *in vitro* study, skin fibroblasts have been reported to have a significant effect over microvascular formation and angiogenesis development from HUVEC [22, 23].

In the entire circulatory system, endothelial cells are lining on the inner layer of the vessels (i.e., tunica intima) and consist of more than 10^13^ cells for approximately 1 kg of vessel [24]. The first culture of endothelial cells was reported by Shibuya around 1930, and then various techniques and sources of endothelial cells were investigated [25]. For *in vitro* studies, endothelial cells from animal or human sources can be used. Endothelial cells from animals include canine jugular endothelial cells and aortic endothelial cells either from bovine (BAEC), porcine (PAOEC), or rat (RAOEC) sources. Human endothelial cells include human umbilical vein (HUVEC), human dermal microvascular (HDMEC), and human vascular (HVEC) [25]. Since endothelial cells from animals can result in immune responses if used in humans, and due to the available sources of endothelial cells, HUVEC have been utilized by many research groups [24, 26].

Heterogeneity between endothelial cells has been reported not only between large vessel-derived cells and those of microvascular origin, but also between organs [24, 25]. For example, aortic endothelial cells are thicker and cover a small area compared to those from human pulmonary artery [27]. Also, endothelial cells from microvascular (e.g., human dermal microvascular endothelial cells (HDMECs)) are elongated, while those from human umbilical vein (HUVEC) are polygonal [27, 28].

Furthermore, endothelial cells derived from arteries are different than those isolated from veins. In arteries, endothelial cells are long and narrow, aligned in the direction of blood flow, and form a continuous endothelium with many tight junctions. Endothelial cells from veins are shorter and wider and not aligned in the direction of blood flow [29]. These heterogeneities reflect their difference in functionality in term of the release of vasoactive substances and their interaction with leucocytes during normal vessel development and wound healing [28, 29].

Some unique molecular markers as well as genes are preferentially expressed by endothelial cells. For example, the expression of CD31 (also known as platelet endothelial cell adhesion molecule-1 or PECAM-1), CD34, and von Willebrand factor (vWF), as well as dil-acetylated LDL uptake has been used in many studies to distinguish endothelial cells from other cell types [29, 30]. Also, cell adhesion molecules (CAM), such as ICAM-1 (intercellular adhesion molecule-1), VCAM-1 (vascular cell adhesion molecule-1), and E- and P-selectins can be used to identify endothelial cells during wound healing and angiogenesis [30, 31].

Other cell-cell adhesive proteins, such as ESAM (endothelial cell selective adhesion molecule), VE-cadherin (vascular endothelial cadherin), and N-cadherin, are known to have a significant in angiogenesis regulation [27, 30]. ESAM is a tight junction molecule that is responsible for the regulation of cellular permeability and for maintaining the polarity of endothelial cells, while VE-cadherin is an adherens junction molecule that plays an important role in regulating cell growth and in the organization of new vessels during angiogenesis [30–32]. Another member of the cadherin family, N-cadherin is localized on the basal side of endothelial cells. During vessel maturation, N-cadherin is in contact with pericytes or smooth muscle cells [32].

Furthermore, the expression of some genes to assess if endothelial cells are undergoing angiogenesis has been suggested. Some genes that are preferentially expressed during endothelial cell sprouting, lumen formation, and capillary network establishment are *HESR-1, notch 1, notch 4,* and *delta 4* [23, 33].

## 3. Vasculogenesis and Angiogenesis

Recent studies on blood microvessels have provided essential information to develop strategies allowing neovascularization. Microvessels can develop through two processes: vasculogenesis and angiogenesis. Both can be potentially applied to vascularize tissue constructs. Although in some papers both terms are often used to mean the same thing and are simply referred to as angiogenesis; the specific role of the microenvironmental factors and the overall mechanisms are different [34].

Vasculogenesis refers to the process of differentiation of endothelial progenitor cells (i.e., mesodermal, mesenchymal, or bone marrow cells) to form new blood vessels. For example, in dermal tissues, vasculogenesis occurs in three main steps: (1) differentiation of mesodermal cells into angioblasts or hemangioblasts; (2) differentiation of angioblasts or hemangioblasts into endothelial cells; (3) the organization of new endothelial cells into a primary capillary plexus [34, 35].

Angiogenesis can occur in two different ways: (1) intussusceptive, the longitudinal splitting of existing vessels, and (2) sprouting angiogenesis, the outgrowth of a new branch from a preexisting vessel [36]. Compared to the intussusceptive angiogenesis that mostly occurs during new organ formation as well as during tumour development, sprouting angiogenesis is relatively well characterized [36]. Sprouting angiogenesis refers to the formation of new capillaries from preexisting blood vessels, which is mainly initiated by a sprouting process [36, 37].

Angiogenesis involves four different stages: (1) endothelial cells interact with their ECM and the underlying basement membrane; (2) they proliferate, migrate, and communicate with each others; (3) they form cell-cell connections and tube-like structures; (4) tube sprouting and remodelling occur to form microvessels containing multicellular lumen [37].

**Figure 1:**
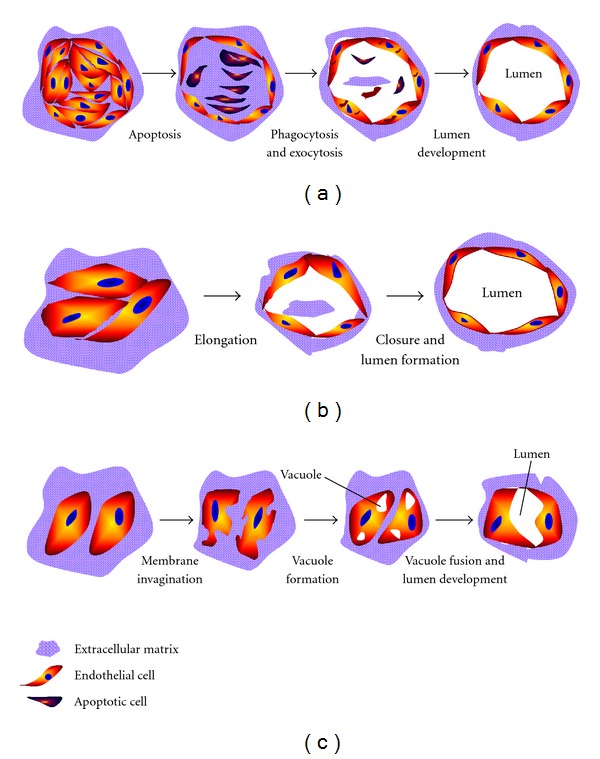
Models of the development of tube-like structures during vasculogenesis.

Angiogenesis as well as the availability of the numerous models now existing has been pioneered about 30 years ago by Folkman and Haudenschild (1980) when they demonstrated that new capillary blood vessels form in tumor progression. They observed that capillary tube formation was initiated from a vacuole structure within the endothelial cells that subsequently develop multicellular capillary lumen [38]. More recently, Kucera et al. (2007) have proposed three models explaining how tube-like structures developed during the vascularization process: (1) cell death and phagocytosis, (2) Wrapping of the spaces around the extracellular matrix, and (3) vacuole formation from the coalescence of intracellular vacuoles. The models are presented on Figure 1, and below are the details [39].

### 3.1. Cell Death and Phagocytosis Model

Some *in vitro* as well as *in vivo* studies of angiogenesis report that lumen formation was associated with apoptotic and cell death events. This model proposed some steps of the vascular lumen formation: endothelial cells organize themselves into a multicellular cord, and cells in the middle of the cord become apoptotic and die [40]. Finally, endothelial cells at the edge phagocytise the apoptotic cells, subsequently forming a multicellular vessel containing lumen [40, 41]. According to this model, dead cells define the lumen of the tube-like structures (see Figure 1(a)). However, this model fails to explain how the tube-like structures continue their formation into a continuous vessel.

### 3.2. Wrapping Spaces around ECM Model

This model mainly suggests that most capillaries are initiated by the elongation of endothelial cells to open multicellular structures. Subsequently, vascular tube can form when the elongated cell sheets are closed, even without vacuole formation (see Figure 1(b)). This concept was initiated by Hirakow and Hiruma (1983), when they studied the development of vascular lumen made of endothelial cells without the evidence of vacuole-like structures [42]. In a recent study, Parker et al. (2004) have shown that the vascular development was initiated by the proliferation and migration of endothelial cells to form a cord-like structure, and vascular cords then undergo tubulogenesis to form vessels containing lumen [43]. However, this model does not explain the polarity of endothelial cells during the development of capillary structures.

### 3.3. Vacuole Formation from the Coalescence of Intracellular Vacuoles

The idea behind this model was proposed by Folkman and Haudenschild (1980) when explaining angiogenesis and lumen formation within 3D constructs. This model involves several steps: formation either of single or multiple vacuole(s) inside the endothelial cells and vacuoles subsequently fuse with each others. Finally, a continuous multicellular lumen will form when two or more cells with their vacuoles adhere to each others, therefore fusing (see Figure 1(c)) [38].

More recent, Davis et al. (2000) have reported that endothelial cell lumens were forming in three-dimensional collagen and fibrin matrices. The authors reported that this process was controlled by the formation and coalescence of intracellular vacuoles and in the absence of cellular junctions [44]. Furthermore, the expression of cytoskeletal regulators controlling various cellular functions of endothelial cells, such as Cdc42 and Rac1 GTPases, were involved in morphogenesis and vascularization processes [44, 45].

Even though this model can explain the role of vacuoles in capillary tube structure and lumen formation, some questions remain, such as how endothelial cells compensate their basal side during the vacuole coalescence and vacuole fusion and how the apical-basal polarity of endothelial cells can be established during tube stabilization [38, 46]. Since none of these three models seem to include all the cellular events observed during vascular tube development, it can thus be hypothesized that all these three models can be combined, in some way, to explain observations made in angiogenesis and vasculogenesis assays [46, 47].

## 4. Promoting Angiogenesis in Three-Dimensional Constructs

During vasculogenesis and angiogenesis development, cell-cell as well as cell-ECM interactions are complex. While the roles of some individual factors during neovascularization have been investigated, the optimum combination between cell and their ECM can significantly vary from one material to another [48]. Therefore, some strategies have been proposed to promote and direct angiogenesis in 3D environments.

Endothelial cells have been cultured in various matrices that have been precoated or not with adhesive matrix proteins or cell binding peptides. Surface chemistry and the immobilization mode can affect protein conformation, orientation and anchorage strength, thus influencing cell behaviour [48]. Formation of capillary-like structures made of endothelial cells depends on the particular biological environment that will direct cell-cell as well as cell-biomaterial adhesion strength, which relies on biochemical and mechanical signals [49]. To date, there are three major strategies that have been used to study how biomaterials properties affect angiogenesis formation: (1) micropatterning, (2) surface modification, and (3) extracellular matrix modifications [48, 49].

### 4.1. Micropatterning

Photolithography, micromoulding, and microprinting are some examples of techniques used in micropatterning. Among them, laser microcontact printing is the most frequently studied [50]. It is a process in which biological molecules are printed directly onto a scaffold surface, and it has been broadly utilized by tissue engineering scientists. For example, a poly(ethylene glycol)-diacrylate (PEGDA) hydrogel, known to resist protein adsorption, has been micro-printed with the cell adhesive ligand, Arg-Gly-Asp-Ser (RGDS) in different concentrations and using different patterns. Endothelial cells were cultured on these RGDS patterns and enhanced tube-like structure formation was observed on 50 *μ*m-wide stripes [51].

In another study, microcontact printing was used to form various patterns of fibronectin molecules on gold. Endothelial cells cultured on these fibronectin patterns formed tubular structures on 10 *μ*m-wide lines of fibronectin [52]. In a different study, when coated with gelatin, 20 *μ*m lines promoted endothelial cells to undergo capillary morphogenesis [53]. However, applying this technique to 3D scaffolds is problematic.

### 4.2. Surface Modification

Proteins such as fibronectin, gelatin, collagens, and fibrinogen, only to name a few, have been used to coat synthetic polymers to increase their surface bioactivity. For example, collagen coating on the surface of electrospun poly(L-lactic acid)-co-poly(*ε*-caprolactone) scaffold increased endothelial cell adhesion and spreading [54, 55]. Collagen type I has been known to increase the number of tube-like structures made of endothelial cells, thus supporting angiogenesis and vasculogenesis in *in vitro* experiments [56]. Furthermore, the proteolytic cleavage of collagen type IV provides an important binding site for endothelial cells undergoing angiogenesis *in vitro* [57].

Gelatin is used by many research groups to pre-coat tissue culture dishes. In tissue engineering, gelatin was used to coat poly(glycolic acid) (PGA) scaffold for the controlled release of some angiogenic growth factors [58]. When PGA scaffolds covered by bFGF- and VEGF-containing gelatin hydrogel were implanted in mice, significant angiogenic effect was observed at the implanted site [59, 60]. Further clinical test to treat diabetic foot ulcer using gelatin matrix to release bFGF confirmed that the matrix allowed better wound healing [60].

Fibronectin has been investigated in many studies and found to enhance endothelial cell adhesion and spreading [61]. Fibronectin also supports the formation of focal adhesion and induces the organization of actin filaments into stress fibre via the interaction with its main receptor, that is, *α*5*β*1 and *α*v*β*3 [61, 62]. Furthermore, the lack of fibronectin during vascular development caused an abnormality in the vascular formation in mice [63].

Another protein, fibrinogen, has been incorporated in poly(ethylene glycol) (PEG) hydrogels and it found to increase the bioactivity of the scaffold by enhancing endothelial cell and SMC adhesion [64] as well as mesenchymal stem cell adhesion [65]. Furthermore, fibrinogen has many binding sites for endothelial cells. Also, fibrin gel binds many growth factors and bioactive cloth components [65, 66]. Therefore, the use of fibrin(ogen) to modify biomaterials surfaces and to encapsulate cells in tissue engineering applications is of importance.

In other strategies, polymer surfaces can be precoated with cells to increase the bioactivity of the scaffold. For example, expanded poly(tetrafluoroethylene) (ePTFE) was precoated with bladder carcinoma cells before being implanted in mice. This strategy stimulated angiogenesis and neovascularization in ePTFE vascular grafts [67]. In a more recent study, precoated PET fibres with HUVEC allowed the guidance of the angiogenesis process and subsequent micro-vascularization in fibrin. No microvessel structures were observed when uncovered fibres were utilized [68].

### 4.3. ECM Modification

The interaction between cells and their matrix is very complex, since there are many proteins and soluble molecules (including growth factors) present in the ECM. These molecules interact with cells through different cell-binding domains. In blood vessel development, there are approximately 20 angiogenic growth factors, and among them vascular endothelial growth factor (VEGF), basic fibroblast growth factor (bFGF), platelet-derived growth factor (PDGF), and angiopoietin-1 (Agp-1) which are the most frequently studied [69].

In *in vitro* angiogenesis assays, growth factors can be added to the culture media and/or immobilized in the ECM. These strategies have been found to have a significant effect on the development of tube-like structures and on microvessel maturation [70]. For example, endothelial cells cultured in type I collagen gels with culture media supplemented with fibroblast growth factor-2 (FGF-2), vascular endothelial growth factor (VEGF), and phorbol ester formed tubes containing lumens, which have similar structure to microvessels found *in vivo* [69]. In the absence of growth factors and phorbol ester, the vascular tube structures did not form [71]. Other *in vitro* angiogenesis studies showed that growth factors added to culture media can promote sprouting, lumen formation, and better vessel stability [72, 73].

Growth factors are often bound to the ECM and can be released upon interaction with cells [74], as well as immobilized to the ECM that was used to carry out the culture [75]. In this approach, growth factors can be released, as encountered *in vivo*. For example, VEGF was covalently immobilized into type I collagen gel [76]. Using the chicken chorioallantoic membrane (CAM) assay, this collagen matrix was found to induce capillary formation and tissue ingrowth. Also, basic fibroblast growth factor (bFGF) was immobilized into poly(ethylene glycol) (PEG) hydrogels to guide cell alignment and migration [77].

As many cellular processes observed during vasculogenesis require numerous signalling pathways and growth factors, recent research efforts have been focusing on the sequential delivery of multiple growth factors [78]. For example, the sequential delivery of VEGF and PDGF-BB (platelet-derived growth factor-BB) in a controlled-release polymer device made of poly(lactic glycolic) (PLG) (85 : 15 lactide : glycolide) induced a mature vascular network covered by smooth muscle cells [79]. Other results also concluded that one growth factor alone is not sufficient to create mature and stable vasculature [80].

### 4.4. Coculture Systems and Microvessel Maturation

Achieving microvessel maturation and stabilization during blood microvessel development represents another challenge following vascular tube formation by endothelial cells. This is often referred to as angiogenesis remodelling. Angiogenesis remodelling is the process by which primary vascular tubes or immature vessels are modified to form an interconnecting branching network, yielding to microvessel structure stabilization [74, 81]. During this phase, tube-like structures made of endothelial cells and containing lumens recruit other supporting cells, such as pericytes and smooth muscle cells (SMCs), to form a close wall and mature blood microvessels [82].

Furthermore, in the vascular tree, endothelial cells are lining on the inner layer of the vessel wall (tunica intima) and supported by other cell types [81, 82]. While immature vessels consist of tube structures made of endothelial cells, capillaries consist of tubes made of endothelial cells covered by pericytes and a basement membrane [82]. In arterioles and venules, vascular SMC cover large area and are closely associated with the endothelium basement membrane.

Therefore, co-culture systems seem to be appealing to recreate the real microvessel environment and to achieve more mature microvessels. For example, smaller number of vessel-like structures containing lumens was found when endothelial cells were cultured alone in PLLA-PLGA (50 : 50) scaffolds, compared to samples cocultured with fibroblasts [83]. Vascularized engineered skeletal muscle tissue constructs have been successfully produced by Levenberg et al. using a multiculture system of myoblasts (progenitor cells of the muscle cells), fibroblasts, and endothelial cells into this PLLA-PLGA scaffold [83, 84]. In more recent result, coculturing HUVECs with skin fibroblast was significantly improved the maturation of microvessels structure in a 3D environment [85, 86].

## 5. Bioreactors to Support Vascularization

Many tissue engineering scientists believe that the next generation of functional tissue replacements truly needs the use of bioreactors, in which the culture conditions (e.g., pH, temperature, O_2_ concentration, pressure, pulsation, nutrient transfer, and waste removal, etc.) can be adjusted and studied to find the optimal mechanical, chemical, and biological stimuli for a given application [87]. In addition, bioreactor operations will provide a rational basis for the structural and functional design of engineered tissues for the use as model systems to reduce time of innovation, discovery, and production in biological and clinical research [87, 88]. Also, the use of bioreactors should accelerate the development, evaluation, and delivery of engineered tissue products to patients [89].

To date, there are various types of bioreactors that have been designed and tested and these differ from the applications involved. For example, bioreactors have been used to support the growth of cartilage [90–92], bone [93, 94], cardiac [95, 96], ligament [97], and heart valve [98] tissues.

Furthermore, there are some types of bioreactors that can be used in large tissue cultures, and these include the continuous stirred-tank reactor (CSTR), hollow fiber reactors, and the slow turning lateral vessel (STLV) reactor [88], only to name a few. CSTR reactors are designed to mix oxygen and nutrients within the culture medium and to reduce the boundary layer at the scaffold surface [99]. Hollow fibre reactors consist of a chamber perfused with semipermeable fibres filled with culture medium. Often, cells are located in the extracapillary space of the chamber. In the past years, this type of reactor was used to produce proteins made by mammalian cells, and to expand mammalian cells *in vitro* [100, 101]. STLV reactor was proposed by NASA (National Aeronautics and Space Administration) at the Johnson Space Center (USA) for earth-based as well as space experiments [101].

In the context of vascularization and blood microvessel development, ideally the bioreactor should enable to control basic environmental parameters such as the dissolved oxygen concentration, pH, and temperature, as well as tissue factors including cellular structures and function [102]. For example, the system should allow the transport of nutrients and oxygen from the reservoir to the cell location within the scaffold. As it has been long known, the maximum tissue thickness achievable by diffusive transport alone is approximately 1 mm. The optimization of scaffold material properties as well as the design of bioreactor chamber is therefore of upmost importance [103].

Bioreactors can also enable control over the mechanical stimulation for cellular guidance inside the scaffold in order to improve tissue vascularization. As endothelial cells are lining the inner surface of blood vessels of the entire capillary and circulatory system, they experience fluid shear stress and dynamic flow conditions [103]. Therefore, the use of a dynamic culture system can allow some cell mechanical stimulation prior to the use of the grown tissue construct [103, 104].

Dynamic culture parameters such as pulsation, pressure, and flow rate that mimic *in vivo* conditions often result in better cellular organization as well as mechanical properties of the tissue constructs when compared to constructs cultured in static environment [105, 106]. To date, several studies of endothelial cell monolayers have revealed that cell orientation reflects the direction of the flow [107, 108]. Also, in endothelial cells, shear stress-increased VEGF, and PDGF-BB expression compared to those cultured in static conditions [109]. In addition, pulsatile flow can improve structural organization of SMC and this is an essential step in the vascularization process [110, 111].

## 6. Concluding Remarks

Over the past years, significant advances in tissue engineering research have provided hope for the commercial availability of human bioartificial organs. More simple engineered tissue substitutes (e.g., skin, bone, and cartilage) have been successfully applied and implanted, while for more complex and vascularized constructs, many problems still remain before tissue substitutes can be available. Consequently, strategies to develop engineered tissue constructs that enhance vascularization inside become important. This will provide an improvement of oxygen and nutrient transfer as well as waste removal that could allow production of thicker tissue substitutes as well as artificial organs. Tissue engineering research relies on the increasing knowledge of angiogenesis and vasculogenesis mechanisms occurring during capillary tube formation and blood microvessel development. Furthermore, to overcome the bottleneck of the complex interplay between various factors influencing tissue vascularization, the use of bioreactor system is necessary.
